# Oral Pregabalin in Cardiac Surgery: A Systematic Review and Meta-Analysis of Randomized Controlled Trials

**DOI:** 10.1155/2021/8835891

**Published:** 2021-03-03

**Authors:** Xian-xue Wang, Jing Dai, Xing-guo Hu, Ai-guo Zhou, Dao-bo Pan

**Affiliations:** ^1^Department of Anesthesiology of the First People's Hospital of Changde City, Changde, Hunan, China; ^2^Department of Anesthesiology of the Taoyuan County People's Hospital, Changde, Hunan, China

## Abstract

**Background:**

Pregabalin has received wide clinical attention as a new type of analgesic. We undertake a systematic review and meta-analysis to evaluate the effect of pregabalin on postoperative pain in patients undergoing cardiac surgery.

**Methods:**

We searched PubMed, Embase, and Cochrane Library (from inception to July 2020) for eligible studies. The primary outcomes were the total morphine consumption at 24 h. A secondary outcome was intraoperative fentanyl consumption, extubation time postoperative, and length of stay in hospital. We calculated pooled weighted mean difference (WMD) or odds ratio (OR) and 95% CIs using random- or fixed-effects models.

**Results:**

Seven trials involving 463 patients were listed. Meta-analysis showed that the total morphine consumption at 24 h in the pregabalin group was significantly less than the control group (WMD: -5.44, 95% CI: -10.42–0.46, *P* = 0.03). We found that there is no significant difference between the two groups in intraoperative fentanyl consumption. Compared with the control group, the length of stay in hospital in the pregabalin group was significantly shorter (WMD = -0.87, 95% CI: -1.42−0.32, *P* = 0.002). And we found that there were no significant differences between the two groups in extubation time (WMD: 17.24, 95% CI: -24.36−58.84, *P* = 0.42).

**Conclusions:**

Oral pregabalin for cardiac surgery patients can effectively reduce the patient's 24-hour morphine consumption after surgery, shorten the patient's hospital stay, and is more conducive to early postoperative recovery.

## 1. Introduction

In recent years, the application scope of enhanced recovery after surgery(ERAS) has become more and more extensive, and at the same time, it has been applied in many clinical departments including cardiothoracic surgery and has received good results [1, 2]. Among them, pain management is an important aspect and requires anesthesiologists to actively participate in this important stage of postoperative recovery patients to improve the quality of postoperative recovery. Effective postoperative pain management can greatly improve the quality of patients, shorten the length of hospital stay, accelerate clinical turnover, and enable the rational allocation of clinical resources [3, 4]. Currently, multimodal analgesia is mostly used for postoperative pain treatment. In addition to traditional opioids, nonsteroidal anti-inflammatory drugs, gabapentin, pregabalin, and other drugs are involved.

Pregabalin, one of the options of analgesics, was initially used clinically as an antiepileptic drug. Depend on its central analgesic effect, it has been widely favored by clinicians in recent years due to its effective analgesic effect. Pregabalin is often used clinically to assist analgesia after surgery [5, 6]. In orthopedic surgery, pregabalin showed a good analgesic effect, which can significantly reduce the patient's postoperative VAS score and reduce the use of postoperative analgesics [7, 8]. In gynecological surgery, through systematic evaluation and analysis, the application of pregabalin can effectively reduce the postoperative pain score of patients with gynecological surgery and reduce the use of analgesics without increasing the incidence of adverse reactions in patients [9, 10]. Most patients undergoing heart surgery suffer from incision pain. The application of pregabalin in these patients has gradually increased, but the results of the studies are not the same. At the same time, there is no systematic review of pregabalin in cardiac surgery. Based on this, the research team included a randomized controlled study on the application of pregabalin in cardiac surgery to comprehensively evaluate its impact on postoperative pain in cardiac surgery patients.

## 2. Methods

This systematic review was conducted according to the guidelines of the Preferred Reporting Items for Systematic Reviews and Meta-Analyses (PRISMA) [11]. We prospectively registered our system review at PROSPERO (registration number: CRD42020203862). We followed the methods of Wang et al. 2017 [12].

### 2.1. Data Sources and Search Strategy

PubMed, Embase, and Cochrane library databases were searched from inception to July 2020 for relevant studies investigating the effect of pregabalin in cardiac surgery. The following search terms were used: Pregabalin, Cardiac surgery, “Coronary artery bypass”, Valve replacement, “Ventricular septal defect repair”, and “Atrial septal defect repair.” A hand search in reference sections of included trials, published meta-analyses, and relevant review articles was conducted to identify additional articles. If duplicated data were presented in several studies, only the most recent, largest, or most complete study was included.

### 2.2. Study Selection

Original studies included were based on PICOS (patient, intervention, comparison, outcome and study design) as the following: (a) *P*: adult patients undergoing cardiac surgery; (b) *I* and *C*: pregabalin and blank control, respectively; (c) *O*: opioid use, length of hospital stay, and extubation time; and (d) *S*: only randomized controlled trials (RCTs) were included. Only English was set.

### 2.3. Data Extraction

Characteristics of patients and trials design were also recorded. If the data mentioned above were unavailable in the article, the corresponding authors were contacted for missing information. All data were independently extracted using a standard data collection form by 2 reviewers (XX Wang and HJ Guo), and then the collected data were checked and entered into Review Manager analyses software (RevMan) Version 5.3. All discrepancies were checked, and a consensus was reached by discussion with a third author (XG Hu) involved. A record of reasons for excluding studies was kept.

### 2.4. Assessment of Study Quality

A critical evaluation of the included studies quality was performed by 2 reviewers (XX Wang and HJ Guo) by using a 5-point Jadad scale [13]. The main categories consisted of the following 5 items: “was the study described as randomized? (1)”, “was the method used to generate the sequence of randomization described and appropriate (random numbers, computer-generated, etc)?” (1) “was the study described as double-blind? (1)”, “was the method of double-blinding described and appropriate (identical placebo, active placebo, dummy, etc)? (1)”, and “was there a description of withdrawals and drop-outs? (1).” A score of 4 to 5 was considered a high methodological quality.

### 2.5. Assessment of Risk of Bias

Two reviewers (XX Wang and HJ Guo) independently evaluated the risk of bias according to the recommendations from the Cochrane Collaboration [14]. The main categories consisted of random sequence generation, allocation concealment, blinding of participants and personnel, blinding of outcome assessment, incomplete outcome data, and selective reporting and other bias. Each domain was assessed to “high risk,” “low risk,” or “unclear risk.” Namely, the judgment was “low risk” for the item with sufficient and correct information. And the judgment was “high risk” for the item reported incorrectly. If the information of the item was insufficient or unsanctioned, the judgment was “unclear risk.” An “unclear risk” judgment should also be made if the item was reported, but the risk of bias is unknown. The disagreement was solved by a senior reviewer (XG Hu).

### 2.6. Statistical Analysis

Risk ratio (RR) with 95% CI was used as a common measure of the effect between spinal anesthesia and general anesthesia. The *I*^2^ value was used to estimate statistical heterogeneity. When *I*^2^ < 50%, heterogeneity could be accepted, and the fixed-effects model was adopted. Otherwise, the randomized-effects model was adopted and sensitivity analysis used. Whenever heterogeneity was present, several sensitivity analyses were carried out to identify potential sources. We also investigated the influence of a single study on the overall pooled estimate by omitting one study in each turn. Owing to the limited number (below 10) of studies included in each analysis, publication bias was not assessed. A *P* value < 0.05 was considered statistically significant. Risk-of-bias assessment was conducted by using Review Manager, version 5.3 (The Cochrane Collaboration, Software Update, Oxford, UK). Power analyses of individual studies and meta-analysis were all conducted by the software, version 4.1.0.

## 3. Results

### 3.1. Identification of Eligible Studies

**Figure 1:**
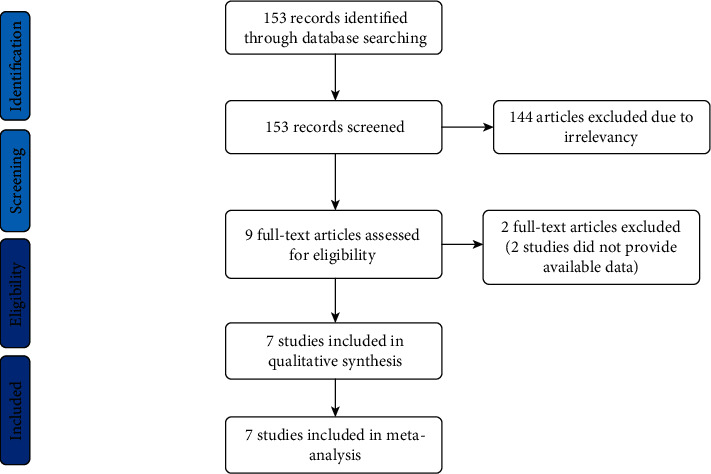
Flow diagram of search strategy and study selection.

A total of 153 potentially relevant abstracts were identified. After reading the abstracts, nine publications seemed to meet the inclusion criteria. For the remaining 9 articles, two of them were excluded for the reason: meta-analysis [15] and commentary [16]. Finally, the remained 7 studies [17–23] with available data met our selection criteria and were included in the systematic review. The flow diagram of search strategy and study selection was presented in Figure 1.

### 3.2. Study Characteristics

The characteristics of all included studies were presented in Table 1. All were adult patients undergoing cardiac surgery. The quality of the included studies was assessed by the Jadad score. All the studies have high Jadad score (range from 4 to 5). Publication bias was not assessed.

These studies were published between 2011 and 2019. The sample size of included studies ranged from 40 to 100. All were randomized controlled trials, and primary end points were morphine consumption, intraoperative fentanyl consumption, extubation time, and length of stay in hospital. All were cardiac surgery, and four of the studies were coronary artery bypass grafting [17–20]. The application of pregabalin is oral, and application time point is slightly different. No significant side effects were found on pregabalin applied to cardiac surgery patients (Table 1).

#### 3.2.1. Total Morphine Consumption at 24 h

**Figure 2:**
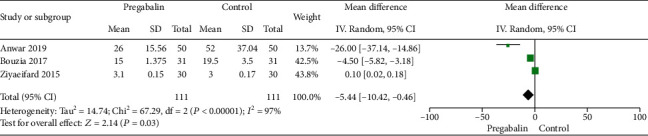
Total morphine consumption at 24 h.

Three studies have examined the total morphine consumption at 24 h [20–22]. Heterogeneity was noted among the three studies (*I*^2^ = 97%; *P* < 0.00001), and a randomized-effects model was selected. Compared with the control group, the total morphine consumption at 24 h in the pregabalin group was significantly lower (WMD: -5.44, 95% CI: -10.42−0.46, *P* = 0.03) (Figure 2).

#### 3.2.2. Intraoperative Fentanyl Consumption

**Figure 3:**
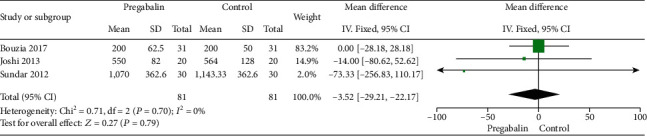
Intraoperative fentanyl consumption between two groups.

Three studies are [17, 19, 22] compared intraoperative fentanyl consumption in the pregabalin group and control group. There were no heterogeneity among the studies (*I*^2^ = 0%; *P* = 0.70), and a fixed-effects model was chosen. After examined the studies by meta-analysis, we found that there were no significantly different between two groups of intraoperative fentanyl consumption (WMD: -3.52, 95% CI: -29.21−22.17, *P* = 0.79) (Figure 3).

#### 3.2.3. Extubation Time Postoperative

**Figure 4:**
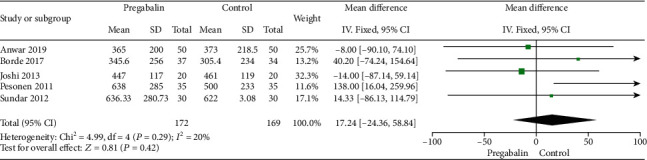
Extubation time after operation.

Five studies [17–19, 21, 23] with a total of 341 patients reported the extubation time of cardiac surgery patients. Heterogeneity among the studies could be accepted (*I*^2^ = 20%; *P* = 0.29), and a fixed-effects model was selected. When compared with the control group, pregabalin was not associated with a significant reduction in extubation time (WMD: 17.24, 95% CI: -24.36−58.84, *P* = 0.42) (Figure 4).

#### 3.2.4. Length of Stay in Hospital

**Figure 5:**
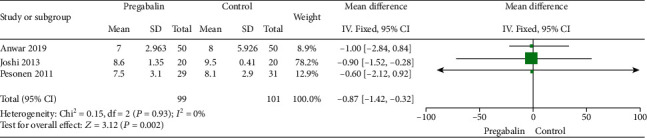
Length of stay in hospital.

Three studies are [17, 21, 23] compared with length of stay in hospital in two groups. There were no heterogeneity among the studies (*I*^2^ = 0%; *P* = 0.93), and a fixed-effects model was chosen. The results suggest that the length of stay in hospital in the pregabalin group was significantly shorter than the control group (WMD = -0.87, 95% CI: -1.42−0.32, *P* = 0.002) (Figure 5).

### 3.3. The Basic Conditions of the Two Groups of Patients

We conducted a comparative analysis of the basic preoperative conditions of the two groups of patients included in the study. Through a systematic evaluation, we found that the preoperative age, height, weight, incidence of hypertension, and diabetes between the two groups were not statistically significant. The results were as follows: (WMD =0.71, 95% CI: -1.10−2.52, *P* = 0.44), (WMD = −1.16, 95% CI: -3.31−0.99, *P* = 0.29), (WMD = −0.26, 95% CI: -2.02−1.50, *P* = 0.18), (OR = 1.40, 95% CI: 0.85−2.30, *P* = 0.18), and (OR = 0.80, 95% CI: 0.47-1.37, *P* = 0.42). There was no statistically significant difference between the two groups of patients in the number of four antihypertensive drugs used, respectively: beta-blocks (OR = 0.67, 95% CI: 0.36-1.27, *P* = 0.23), Ca channel antagonist (OR = 1.13, 95% CI: 0.57-2.26, *P* = 0.72), ACE inhibitors (OR = 0.85, 95% CI: 0.50-1.43, *P* = 0.53), and diuretics (OR = 1.13, 95% CI: 0.57-2.24, *P* = 0.73) (Table 2).

## 4. Discussion

Postoperative pain is part of the common complications in heart surgery patients. The occurrence of pain is affected by many factors; so, a reasonable choice of analgesic drugs can achieve a good postoperative analgesic effect. Based on a meta-analysis of the included literature, this article found that oral pregabalin during the perioperative of cardiac surgery can significantly reduce the 24-hour postoperative morphine consumption and shorten the patient's hospital stay. However, the intraoperative fentanyl usage and extubation time postoperative in patients who used pregabalin did not show an advantage.

After heart surgery, the incision site often exhibits severe pain and long-lasting. Intense pain can cause changes in the patient's neuroendocrine and circulatory system, which will hinder early postoperative recovery, extend the patient's hospital stay, and increase the economic burden of patients. Therefore, effective analgesia after cardiac surgery can effectively improve the prognosis and promote rapid recovery of patients after surgery, which has important clinical significance. Pregabalin, as a new structural analog of gamma-aminobutyric acid (GABA), was used in clinical practice as an antiepileptic drug in the early stage and gradually began to be used in patients with pathological pain [24]. In recent years, more and more clinicians have found that pregabalin also plays a significant advantage in postoperative pain. Pregabalin mainly combines the *α*2-*δ* subunits of voltage-gated calcium channels to reduce the excitability of nerve synapses and reduces the release of various excitatory neurotransmitters, thereby inhibiting patients with hyperalgesia [25, 26]. This study analyzed the included literature and found that a total of 3 high-quality pieces of literature reported the difference in morphine usage in the pregabalin group and the control group at 24 hours postoperative. A synthetic analysis of the data of these 3 kinds of literature found that the use of pregabalin can significantly reduce the consumption of morphine in the 24-hour postoperative period, which also reflects that the perioperative use of pregabalin in cardiac surgery patients can effectively relieve postoperative pain and reduce postoperative opioid consumption. However, during the operation, we did not find the fentanyl consumption in the pregabalin group that was less than that in the control group through a systematic review during the operation. In terms of affecting the patient's hospital stay, there are also three literature reports that the hospital stay in the pregabalin group was significantly shorter than the control group. After a synthetic analysis of the data, it is found that the conclusions of these 3 documents are highly consistent. This also further shows that the postoperative pain of the patient is reduced, which is more conducive to the early postoperative getting out of bed, the early postoperative recovery, and reduce the corresponding economic burden.

This study included a meta-analysis of a randomized controlled study of the effects of pregabalin on the prognosis of different groups of cardiac surgery patients. It is our objective understanding of the application of pregabalin in cardiac surgery patients. The advantages of pregabalin in cardiac surgery provide a further direction for our next research. On the one hand, we can use this clue to observe its long-term impact on patients and analyze the quality of life; on the other hand, we can expand to other patient application research. The meta-analysis of this study included the baseline data of patients in the literature. It was found that the basic conditions of patients in the included literature were comparable and no significant difference. The heterogeneity between the literature was mostly 0%. Although qualitative exists, they are all relatively small and within acceptable limits. It shows that the consistency of the included literature in this study is good, and the credibility of the literature data is high.

While several limitations should be taken into account, first, the time point and dosage of pregabalin used are different, which affect the comparison of results. Second, most of the literature does not report the relevant scores of postoperative pain, which difficult to directly assess the postoperative pain of the patient, and the postoperative morphine can only be used to indirectly reflect the postoperative pain of the patient. Third, ours were mainly based on studies published in the English language, and bias might be existed. Forth, the sample sizes of individual trials included were small or moderate. Fifth, only selected published studies and many studies were not registered with clinical trials databases; so, the scope of the unpublished literature could not be obtained and may lead to bias.

## 5. Conclusion

Oral pregabalin for cardiac surgery patients can effectively reduce the patient's 24-hour morphine consumption after surgery, shorten the patient's hospital stay, and is more conducive to early postoperative recovery.

## Figures and Tables

**Table 1:** Characteristics of the seven studies included in the meta-analysis.

Author	No. of patients (pregabalin/control)	Country	Surgery	Pregabalin group	Outcomes	Jadad score
Sundar 2012	30/30	India	Coronary artery bypass surgery	Pregabalin 150 mg capsule orally 1 h before surgery	Hemodynamic data, perioperative variables, fentanyl required, visual analog, and Ramsay sedation scores	4
Anwar 2019	50/50	United Kingdom	Cardiac surgery	Pregabalin (150 mg preoperatively and twice daily for 14 postoperative days)	Pain at 3 and 6 months, total morphine consumption, length of stay in cardiac intensive, sedation score, nausea score, and length of stay in hospital	5
Ziyaeifard 2015	30/30	Iran	Coronary artery bypass surgery	150 mg pregabalin capsules	Hemodynamic parameters, morphine consumption, duration of intensive care unit stay, and pain score	4
Pesonen 2011	35/35	Finland	Cardiac surgery	150 mg of pregabalin before operation and 75 mg of pregabalin twice daily for 5 postoperative days	MMSE score, RASS score, CAM-ICU scores, P-creatinine, atrial fibrillation, postoperative pain, nausea, and vomiting	4
Bouzia 2017	31/31	Greece	Cardiac surgery	Pregabalin 150 mg	VRS, morphine, vomiting, intraoperative fentanyl/remifentanil, use of analgesics at 3 months, and sleep disturbance at 3 months	4
Joshi 2013	20/20	India	Coronary artery bypass surgery	Pregabalin capsules (75 mg) were given every 12 h for 2 postoperative days	Pain characteristics, RASS scale	4
Borde 2017	37/34	India	Coronary artery bypass surgery	Pregabalin, 150 mg capsule orally, 1 hour before surgery and 2 days postoperatively	Perioperative hemodynamic parameters, QoR-40 dimensions and global score, intravenous fluids, intravenous fentanyl, and durationof surgery	3

**Table 2:** Subgroup analysis preoperative basic information of two groups.

Variable	Number of studies	WMD/OR(95% CI)	*I* ^2^	Effects models	*P* value
Age	6	0.71 (-1.10-2.52)	0%	Fixed-effects models	0.44
Weight	6	-1.16 (-3.31-0.99)	0%	Fixed-effects models	0.29
Height	5	-0.26 (-2.02-1.50)	15%	Fixed-effects models	0.80
Hypertension	5	1.40 (0.85-2.30)	47%	Fixed-effects models	0.18
Diabetes	4	0.80 (0.47-1.37)	0%	Fixed-effects models	0.42
Antihypertensive drugs preoperative					
Beta-blocks	4	0.67 (0.36-1.27)	0%	Fixed-effects models	0.23
Ca channel antagonist	3	1.13 (0.57-2.26)	0%	Fixed-effects models	0.72
ACE inhibitors	4	0.85 (0.50-1.43)	0%	Fixed-effects models	0.53
Diuretics	3	1.13 (0.57-2.24)	0%	Fixed-effects models	0.73

## Data Availability

No additional unpublished data are available.
